# Percutaneous Transaxillary Impella Device Placement Resulting in Iatrogenic Subclavian Artery Pseudoaneurysm

**DOI:** 10.7759/cureus.40082

**Published:** 2023-06-07

**Authors:** Inderpal Singh, Jordan Swisher, Theodore Schreiber

**Affiliations:** 1 Internal Medicine, Ascension St. John Hospital, Detroit, USA; 2 Cardiology, Ascension St. John Hospital, Detroit, USA

**Keywords:** st-elevation myocardial infarction (stemi), high risk pci, cardiogenic shock, subclavian artery pseudoaneurysm, impella cp

## Abstract

Subclavian artery pseudoaneurysm (PSA) is a rare complication arising from transaxillary Impella device placement during high-risk percutaneous coronary intervention (PCI). Despite the increasing prevalence of Impella use, literature addressing this complication is scarce. This case emphasizes the limited existing evidence on subclavian artery PSA and highlights the importance of recognizing it as a potential risk. With high-risk PCI and Impella use gaining popularity, understanding this complication is crucial for early detection and appropriate management. A 62-year-old male with a past medical history of type II diabetes mellitus, peripheral artery disease, hypertension, and chronic tobacco use presents with recurrent episodes of exertional chest pain and dyspnea. Initial workup with an electrocardiogram showed ST-segment elevations in the anteroseptal leads. The patient underwent right- and left-sided cardiac catheterization, which revealed severe stenosis of the left anterior descending artery and findings of cardiogenic shock. The patient required mechanical circulatory support with a percutaneous left ventricular assist device during the procedure; this was placed via transaxillary approach due to the patient having peripheral artery disease in bilateral femoral arteries. The patient had a complicated clinical course, but the patient's clinical picture slowly improved, and the percutaneous left ventricular assist device was removed. Roughly six weeks after the removal of the device, the patient developed a large fluid collection in the chest wall anterior to the left shoulder. Imaging revealed a ruptured left distal subclavian artery PSA. The patient was promptly taken to the catheterization laboratory and a covered stent was deployed over the site of the PSA. Repeat angiography revealed brisk flow through the left subclavian artery into the axillary artery with no extravasation into the chest wall.

## Introduction

With the rise in the use of high-risk percutaneous coronary intervention (PCI), percutaneous left ventricular (LV) assist devices are used to support patients with complex coronary artery disease and hemodynamic compromise, such as cardiogenic shock and severely depressed LV function [1]. Placement of these devices is commonly inserted percutaneously through the femoral artery, but in patients with lower extremity peripheral artery disease, alternative approaches for placement are necessary [2]. Here, we present a case of distal left subclavian artery pseudoaneurysm (PSA) formation due to insertion of a percutaneous transaxillary LV assist device.

## Case presentation

A 62-year-old male with a past medical history of type II diabetes mellitus, peripheral artery disease, hypertension, and chronic tobacco use presents with recurrent episodes of exertional chest pain and dyspnea for one month. Vital signs on arrival were significant, with a respiratory rate of 24 breaths per minute and a blood pressure of 162/104 mmHg. The patient had an oxygen saturation of 97% in room air. Physical exam was negative for any cardiac murmurs, rubs, or gallops upon auscultation of the precordium. Lung sounds were clear to auscultation bilaterally, and there was no evidence of peripheral edema in the lower extremities. Initial laboratory analysis was significant for an elevated troponin T level of 18.33 ng/mL and a serum creatinine of 0.61 mg/dL. Initial electrocardiogram was done and showed ST-segment elevations in the anteroseptal leads. The patient rapidly decompensated and became hypotensive with a blood pressure of 85/60 mmHg within six to eight hours after initial presentation.

**Figure 1:**
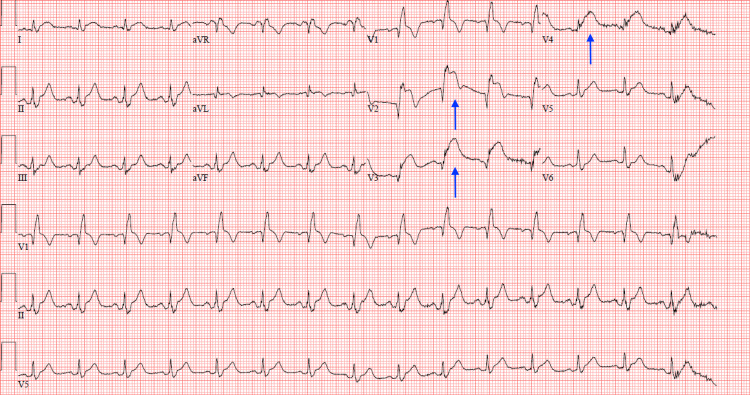
12-lead electrocardiogram showing ST-segment elevations in leads V2, V3, and V4 (blue arrows).

Further workup with an electrocardiogram was done and showed ST-segment elevations in the anteroseptal leads (Figure 1).

The patient was transferred to an outside facility for planned right and left heart catheterization. The patient was transported to a PCI-capable facility and underwent the procedure in less than 120 minutes. Right heart catheterization (RHC) demonstrated elevated pressures in the right atrium and right ventricle. Pulmonary artery pressure and pulmonary capillary wedge pressure were also seen to be elevated. The results of RHC are outlined in Table 1.

**Table 1:** Results of RHC showing signs of LV systolic dysfunction with elevated right atrial, right ventricular, pulmonary artery, and pulmonary capillary wedge pressures. RHC: right heart catheterization; LV: left ventricular.

Test	Result (mean pressure)	Reference range
Right atrial pressure	19/19 (18)	Mean 0-5 mmHg
Right ventricular pressure	35/20 (28)	Systolic 20-25 mmHg, diastolic 0-5 mmHg
Pulmonary artery pressure	43/16 (27)	Mean 9-16 mmHg
Pulmonary capillary wedge pressure	15/12 (7)	Mean 0-5 mmHg

Right-sided cardiac catheterization also revealed a decreased cardiac output and index indicative of severe LV systolic dysfunction and cardiogenic shock requiring mechanical circulatory support with an Impella CP LV assist device (Abiomed Inc, Danvers, MA, United States). The Impella CP device was placed percutaneously through the left axillary artery with fluoroscopic guidance as the patient had severe peripheral artery disease in bilateral femoral arteries as seen on angiography. Percutaneous transluminal coronary angiography revealed 95% stenosis of the proximal left anterior descending (LAD) artery. PCI of the LAD lesion was done with balloon angioplasty, as a 2.5 mm x 30 mm Trek RX balloon (Abbott Cardiovascular, Plymouth, MN, United States) was advanced across the lesion and deployed with a single inflation at 9 atm. A 3.0 mm x 38 mm Xience Skypoint drug-eluting stent (Abbott Cardiovascular, Plymouth, MN, United States) was deployed across the lesion with a single inflation at 12 atm soon thereafter. Upon completion of the procedure, the patient was transferred to the cardiovascular intensive care unit for further monitoring and care.

**Figure 2:**
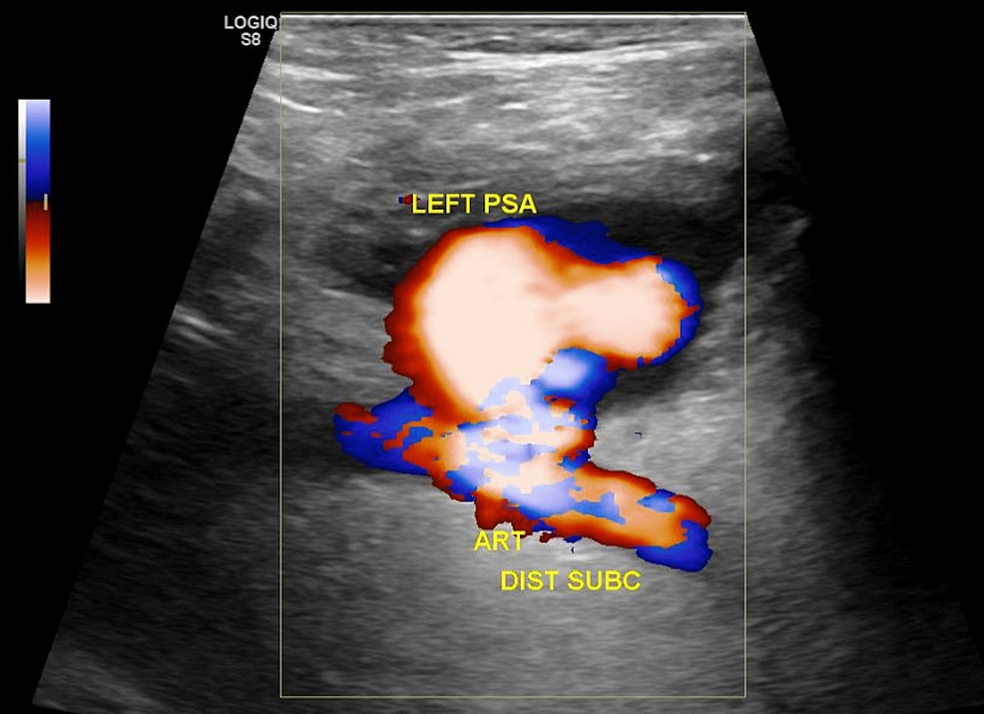
Arterial duplex ultrasound of the left upper extremity showing a distal subclavian artery PSA measuring 3.8 x 6.5 cm with a neck base of 1.2 cm. PSA: pseudoaneurysm.

**Figure 3:**
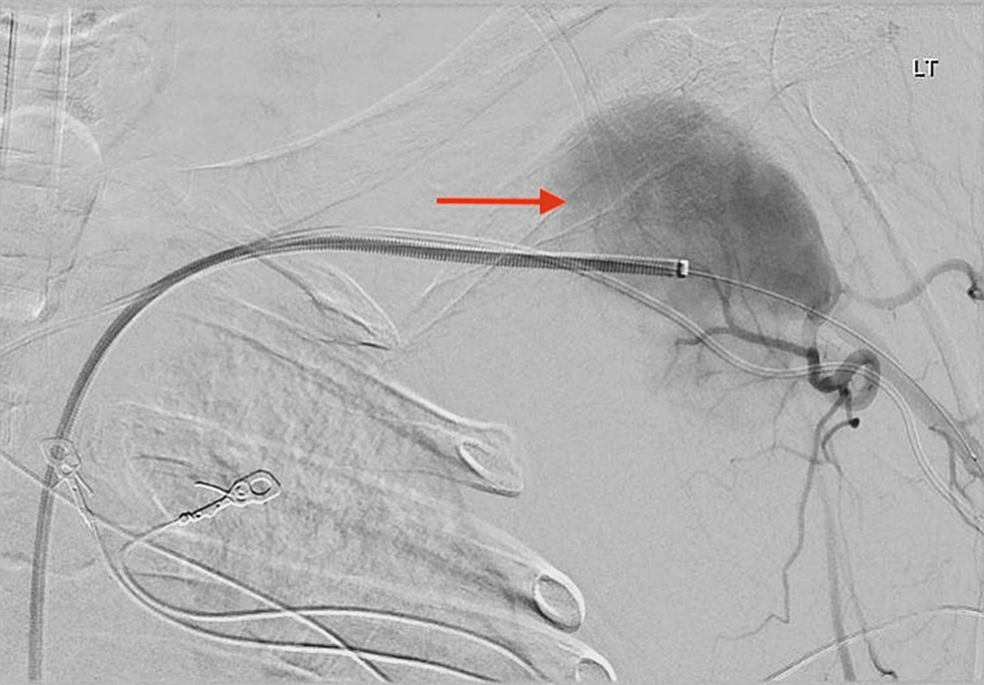
Angiogram of the left subclavian artery showing the distal left subclavian artery PSA with active extravasation prior to stent placement (red arrow). PSA: pseudoaneurysm.

**Figure 4:**
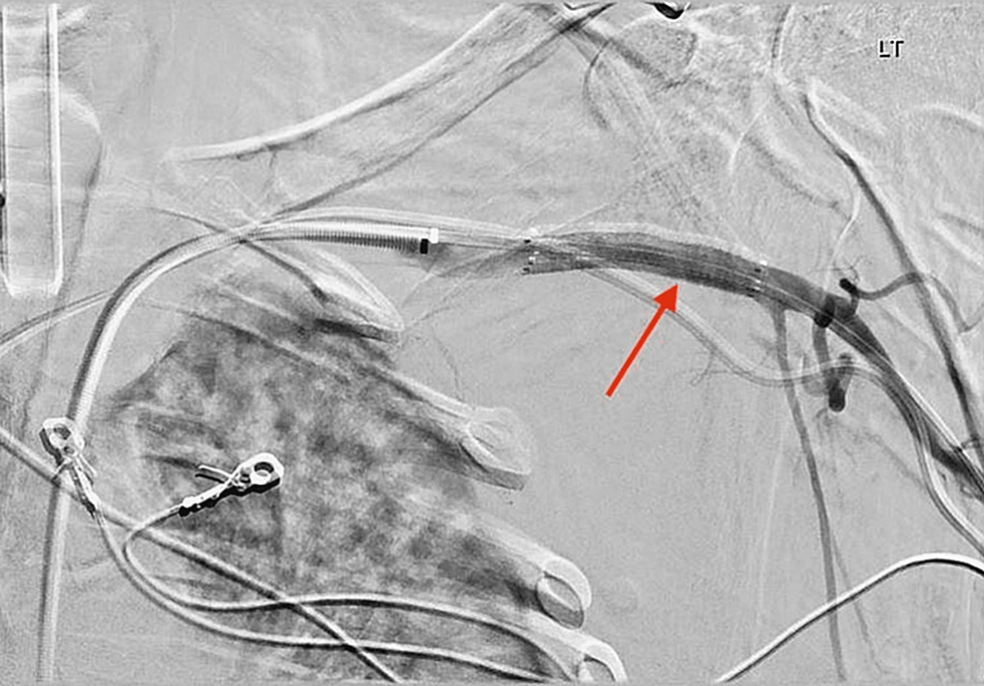
Angiogram of the left subclavian artery showing exclusion of the distal left subclavian artery PSA after an 8.0 mm x 50 mm Viabahn covered stent placement (red arrow). PSA: pseudoaneurysm.

The patient's clinical picture and invasive hemodynamic measurements, including cardiac output and index, improved over the course of the inpatient stay. The patient was brought to the catheterization laboratory, and the Impella CP device was subsequently removed. During the procedure, a left subclavian artery angiogram was performed, which revealed a small subclavian artery dissection. An 8.0 x 40 mm occlusive balloon was inflated to 2 atm for 90 seconds at the site of dissection and adequate hemostasis was achieved. Roughly six weeks after the removal of the Impella CP device, the patient developed a large fluid collection anterior to the left shoulder. Imaging, including a computed tomography scan of the thorax and arterial duplex ultrasound of the left upper extremity, showed findings of a left distal subclavian artery PSA measuring 3.8 x 6.5 cm with a neck base of 1.2 cm (Figure 2). An angiogram of the left subclavian artery through the left femoral artery approach was performed, which showed findings of a large, ruptured PSA with significant extravasation into the chest wall (Figure 3). An 8.0 mm x 50 mm Viabahn covered stent was deployed over the site of the PSA, followed by balloon angioplasty, resulting in brisk flow from the left subclavian artery to the left axillary and brachial arteries with no further extravasation (Figure 4). The extravasated blood from the PSA was aspirated, yielding 300 mL with post-procedure imaging showing a significant decrease in chest wall fluid collection. The patient had no further complications or symptoms, and perfusion in the left upper extremity remained intact after the placement of the covered stent.

## Discussion

Percutaneous LV assist devices have been seen to have an increased role and utility in high-risk interventional cardiac procedures [3]. In patients undergoing high-risk PCI, the PROTECT II study showed a significant decrease in the rates of major adverse events in patients supported with Impella 2.5 compared to patients supported with an intra-aortic balloon pump at 90 days post-procedure [4]. These devices are generally inserted percutaneously through the femoral artery under fluoroscopic guidance with eventual placement in the LV cavity. Transfemoral access may not be suitable for select patients with severe peripheral artery disease of the lower extremities, necessitating alternative approaches for placement [2]. Transaxillary access is an alternative to the transfemoral approach, which has potential benefits of early mobilization and decreased risk of procedure-associated infection [5].

Many vascular complications after percutaneous placement of a LV assist device have been described in the literature. Through the transfemoral approach, common complications include the development of wound hematomas and PSA, with more severe complications being acute limb ischemia requiring amputation [3]. When compared to the transaxillary approach, potential complications may include the development of wound hematomas and injury to adjacent neurovascular structures. More severe complications include the development of hemothorax [5]. The formation of axillary and subclavian artery PSAs due to the transaxillary placement of percutaneous LV assist devices has rarely been reported in the literature. In general, the incidence of iatrogenic PSAs ranges between 0.1% and 6%, with the incidence of upper extremity being less than 2% of all PSAs [6]. As seen in this case, a delayed complication was noted with PSA formation in the distal left subclavian artery after transaxillary placement and removal of an Impella CP device.

Treatment of upper extremity PSA can be done with a surgical or endovascular approach. Endovascular repair is preferred as surgical treatment may be associated with severe complications, such as damage to adjacent neurovascular structures [7]. Endovascular repair reduces the risk of these complications, as was seen in our patient who was treated with the deployment of a covered stent across the lesion. In certain circumstances, specifically if the aneurysmal sac is percutaneously available and if the aneurysmal neck base is less than 3 mm in diameter, ultrasound-guided thrombin injection into the aneurysmal sac can be done for treatment [8]. This approach to treatment has been outlined and discussed in the literature for femoral artery PSAs, although there is a lack of experience with thrombin injections in axillary artery PSAs [9].

## Conclusions

Iatrogenic arterial PSAs are a known but rare complication of percutaneous LV assist device placement during high-risk PCI. Treatment of upper extremity PSAs is generally done endovascularly through the placement of a covered stent across the lesion. There is a paucity of literature describing this complication in the upper extremities, and more data are required to determine the incidence and prognosis of this complication as percutaneous transaxillary approach is adopted during high-risk percutaneous cardiovascular procedures.
